# How Far Have Large Language Models Advanced in Ophthalmology? A Systematic Review of Their Development, Evaluation, and Readiness for Clinical Use

**DOI:** 10.21203/rs.3.rs-8819770/v1

**Published:** 2026-02-10

**Authors:** Hyunjae Kim, Yu Yin, Zhiyuan Cao, Chen Liu, Anran Li, Zhen Chen, Xuguang Ai, Younjoon Chung, Fan Ma, Xueping Peng, Lingfei Qian, Zhenyue Qin, Kalpana Raja, Yang Ren, Weipeng Zhou, Yih-Chung Tham, Emily Y. Chew, Zhiyong Lu, Sophia Y. Wang, Hua Xu, Qingyu Chen

**Affiliations:** 1 Yale University; 2 University of Queensland; 3 National University of Singapore; 4 National Institutes of Health; 5 Stanford University

## Abstract

Large language models (LLMs) are rapidly transforming ophthalmology, with expanding applications in patient care, clinical documentation, and medical education. Recent studies span a wide range of use cases—from early text-only applications to emerging multimodal systems that integrate ophthalmic images to support diagnosis and generate assessment and treatment plans. Amid this rapid progress, it is critical for both researchers and clinicians to stay informed in order to guide responsible development and adoption. However, prior reviews have largely focused on narrow domains such as an inventory of potential use cases or performance on board-style examinations, leaving the broader landscape insufficiently characterized. Key questions remain unanswered: How are LLMs in ophthalmology being developed? What applications and evaluation strategies are being pursued? And which areas are closest to real-world clinical adoption? To date, these aspects have not been comprehensively examined.

In this study, we conducted a systematic review on LLMs in ophthalmology by manually screening 1, 029 studies from PubMed/PMC, Scopus, and Embase published between January 1, 2022, and April 1, 2025, identifying 91 relevant articles. To provide a standardized assessment, we introduced a structured framework that categorizes ophthalmic use cases and stratifies evaluation rigor across five levels of maturity. Each study was manually annotated using 27 structured variables spanning multiple dimensions: scope and purpose (e.g., study aim, ophthalmic subspecialty, input modality); model architecture and training (e.g., backbone LLMs, domain-specific adaptations); evaluation and validation (e.g., target applications, evaluation metrics, level of clinical validation); and resource availability (e.g., model access, licensing, dataset availability). We additionally performed a small-scale, illustrative evaluation of representative emerging models, such as GPT-5.2, gpt-oss-120B, and Gemini 3, to contextualize previously reported results on commonly used ophthalmology tasks.

The results show that most studies focused on general-purpose proprietary models, such as GPT-4 and Gemini, while fewer than 10% introduced domain-specific adaptations for ophthalmology, including only 4% that developed ophthalmology-specific architectures for text-based applications. Multimodal LLMs remain relatively underexplored, with only 23% of studies incorporating imaging data. Evaluation practices reveal a significant translational gap: While 57.1% of studies relied on standard benchmarking and expert review, only 9.9% conducted retrospective validation using real-world clinical data, and just two studies progressed to prospective pilot evaluation. Moreover, although model performance on benchmarks on board-style exams and clinical vignettes has improved with newer model generations, reproducibility and transparency remain limited: only 5.5% of studies released evaluation code, and 33% used publicly available datasets. Finally, we provide a living repository to track the rapid progress of LLMs in ophthalmology for the broader research and clinical community.

## 1 Introduction

The global burden of eye diseases is both substantial and increasing, posing significant public health, social, and economic challenges^1–8^. According to the World Health Organization (WHO), over 2.2 billion people live with visual impairment, including at least 1 billion cases that could have been prevented or remain unaddressed^9, 10^. Global productivity losses attributable to vision impairment are estimated to exceed USD 400 billion annually^3^. Despite advances in ophthalmic diagnostics and treatment, much of this burden remains unavoidable, underscoring the need for scalable, intelligent systems to enhance efficiency and accessibility in eye care.

Large language models (LLMs), a class of artificial intelligence systems trained on vast volumes of text corpora, are emerging as transformative tools across ophthalmic practice and research^11–15^. Recent studies highlight their potential in the field of ophthalmology to streamline documentation and patient communication^16, 17^, assist in diagnostic reasoning and treatment planning^18^, and provide guideline-consistent responses to disease-specific queries^19^. Beyond text-based applications, the advent of multimodal systems that integrate ophthalmic images, such as fundus photographs, optical coherence tomography (OCT) scans, and slit-lamp images, suggests opportunities for unified models that combine linguistic reasoning with visual interpretation, such as automated disease diagnosis and assessment and plan generation^20–22^. These developments have led to a rapid expansion of LLM-based research in ophthalmology, spanning diverse use cases^23–27^.

Researchers and clinicians are essential to stay informed about both recent methodological advances and the real-world potential and limitations of LLMs in ophthalmology. However, existing studies vary widely in their scope and evaluation approaches, ranging from non-clinical, examination-style evaluations to clinical validation using real-world hospital data. Moreover, it remains unclear whether this growing body of work primarily represents an initial phase of applying existing LLMs, or a shift toward more advanced, domain-specific model development. These factors make it difficult to derive a clear understanding of the current state of the field and the clinical readiness of LLMs in ophthalmology. Several pioneering studies have provided preliminary reviews and scoping syntheses of LLMs in ophthalmology^12–14, 28–34^. However, these works have largely focused on specific aspects—such as narrative overviews of potential applications (e.g., applications of ChatGPT)^12–14, 30–32^ and evaluations of LLM performance on board-style examination questions^34^. More importantly, prior reviews have not systematically examined the landscape of LLM development (e.g., methodological innovations and model adaptation strategies) and adoption (e.g., progression from benchmarking to expert evaluation using real-world clinical data and assessments of clinical readiness). Given the rapid pace at which new systems are emerging, existing reviews also risk becoming quickly outdated, highlighting the need for a comprehensive and up-to-date synthesis of recent progress in the field.

The objective of this systematic review is to synthesize recent studies on LLMs in ophthalmology and to assess their methodological development, evaluation practices, and readiness for clinical translation. Specifically, we examined the literature across key dimensions, including clinical scope, use-case categories, evaluation design and depth, model type and modality, training and adaptation methods, data and resource availability, and transparency in reporting practices. We organized ophthalmic use cases into a structured framework and stratified them by a five-level evaluation depth, ranging from benchmark evaluation to clinical trials. These analyses provide a grounded overview of the current state of the field and clarify how existing systems may be used in clinical settings. We also identified recurring limitations and practical gaps that hinder cross-study comparability and real-world deployment, and we highlight priorities for future model development and evaluation. To support progress in this evolving field, we will maintain a publicly accessible website that curates newly published studies through structured summaries and visualized overviews: https://github.com/yale-BIDS-Chen-Lab/ophtho-llm-review. This platform will serve as a living resource for the community to track the latest progress of LLMs in ophthalmology.

## 2 Methods

### 2.1 Search Strategy and Information Sources

We conducted a literature search covering the period from January 1, 2022, to April 1, 2025. Three databases, PubMed/PMC, Scopus, and Embase, were selected based on their widespread use, credibility, and accessibility. The search query was defined as follows:

(“large language model*” OR LLM OR “language model*” OR “foundation* model*” OR GPT OR ChatGPT OR LLaMA OR PaLM OR Gemini OR Mistral OR Falcon OR Claude OR bloom OR qwen OR deepseek OR o1) AND (“Ophthalmology”[Mesh] OR ophthalmology OR eye OR “eye disease*“) AND (pretrain* OR pre-train* OR fine-tun* OR fine-tun* OR “instruction tun*” OR train*) AND (2022/01/01:2025/04/01[dp]) NOT review[pt] NOT systematic[sb] NOT clinical trial[pt] NOT case reports[pt] NOT meta-analysis[pt]

### 2.2 Study Selection

An initial set of articles was identified through systematic searches, followed by a two-stage duplicate removal process. First, exact duplicates were identified by matching normalized titles after lowercasing and removing punctuation. Next, we applied fuzzy sequence matching to capture near-duplicates arising from minor formatting or wording differences, using similarity thresholds of 0.90 for titles and 0.85 for abstracts.

The resulting set of unique articles was then advanced to manual screening. Title and abstract screening were independently conducted by two annotators (Y.Y., Z. Cao), with any discrepancies resolved by a third reviewer (H.K.). Full-text reviews were carried out by a team of 12 annotators (Y.Y., C.L., A.L., Z. Chen, X.A., F.M., X.P., L.Q., Z.Q., K.R., Y.R., W.Z.), with each paper reviewed by two individuals. In cases of disagreement, a third annotator (H.K.) adjudicated the decision.

#### Inclusion criteria

Studies were included if they met all of the following criteria: (1) Primary research articles that were publicly available (published or preprint) within the search window (January 1, 2022 – April 1, 2025). (2) The study primarily developed, evaluated, or utilized LLMs for ophthalmology-related applications. LLMs had to be a central component of the study, either as the main target of evaluation or as a core methodological element, rather than being used solely as a baseline or comparison. (3) Multimodal models were considered eligible when an LLM-based architecture was used as the primary component.

#### Exclusion criteria

Studies were excluded if they met any of the following: (1) Non–primary research articles, including reviews, meta-analyses, editorials, perspectives, correspondence, and study protocols. (2) Studies not published in English. (3) Conference abstracts without a corresponding full-text publication. (4) Multimodal models that did not incorporate LLM architectures as their primary backbone, and vision-only models.

#### Definitions of LLMs

In this review, we operationally define large language models (LLMs) as generative pre-trained language models built on the Transformer architecture^35^. We focus on models developed after the introduction of GPT-3^36^, which marked a paradigm shift toward large-scale, general-purpose language models with emergent few-shot capabilities. LLMs are characterized by their generative nature, large-scale parameterization, extensive pre-training on broad corpora, and substantial computational requirements^37^, distinguishing them from earlier generations of language models. Accordingly, we exclude encoder-only architectures, such as models in the BERT family^38^, as this review focuses on generative modeling paradigms that underpin contemporary general-purpose LLMs.

### 2.3 Data Extraction

**Figure 1:**
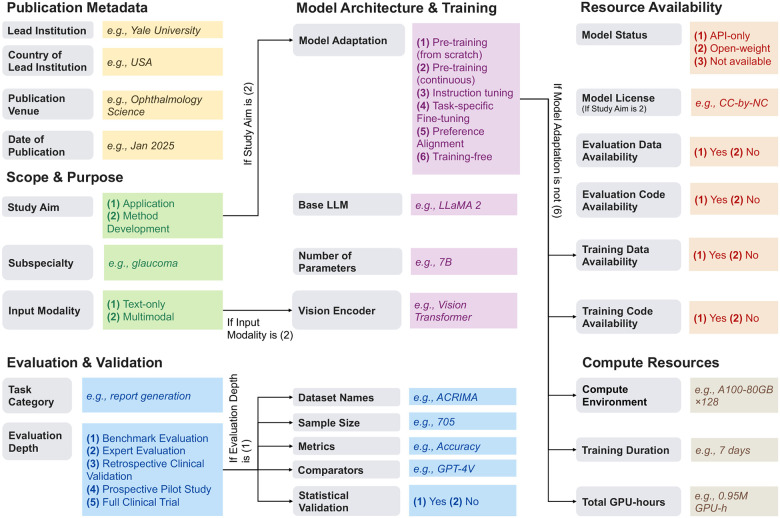
Overview of the 27 variables used for data extraction. The variables consist of both free-text fields (e.g., Lead Institution) and option-based fields (e.g., Study Aim), depending on the nature of the information being captured Some variables are conditionally applied depending on study characteristics; for example, the variable “Vision Encoder” is included only when the condition “If Input Modality is (2) Multimodal” is met. Detailed descriptions of all variables are provided in Extended Data Table 1.

Data extraction was performed by a team of 12 annotators (Y.Y., C.L., A.L., Z. Chen, X.A., F.M., X.P., L.Q., Z.Q., K.R., Y.R., W.Z.). Each article was independently reviewed by two annotators using a standardized form comprising 27 variables across six fields (see Fig. 1). These fields covered:

1. **Publication metadata**: bibliographic and contextual information for each study, including institutional and geographic background, publication date, and publication venue.
2. **Scope and purpose**: the primary study objective and input modality, distinguishing evaluation- or application-oriented studies from methodological contributions, as well as text-only versus multimodal approaches.
3. **Model architecture and training**: the underlying base LLM and vision encoder, reported model scale where available, and the training or adaptation strategies employed, including pre-training, instruction tuning, task-specific fine-tuning, and preference alignment.
4. **Evaluation and validation**: how LLMs were assessed in each study, including the ophthalmic task addressed and the level of evaluation rigor, from benchmark-based testing (including datasets, metrics, comparators, statistical validation) to expert review and clinical validation.
5. **Resource availability**: the accessibility and transparency of models and data, including model release status and licensing, availability of training and evaluation data, and availability of training and evaluation code.
6. **Computational resources**: reported information on the computational environment and training cost, including training duration and total GPU usage where available.

Among 27 variables, ten variables involved selection from predefined options, while others required free-text input. To ensure harmonization, two annotators (Y.Y. and Z. Cao) manually reviewed and standardized the free-text responses. Z. Cao was responsible for assigning the “task category” and “evaluation depth, ” while Y.Y. was responsible for all remaining variables. Discrepancies identified during this normalization process were adjudicated by a third reviewer (H.K.). Detailed definitions and annotation guidelines for each variable are provided in Extended Data Table 1.

## 3 Results

### 3.1 Study Inclusion

**Figure 2:**
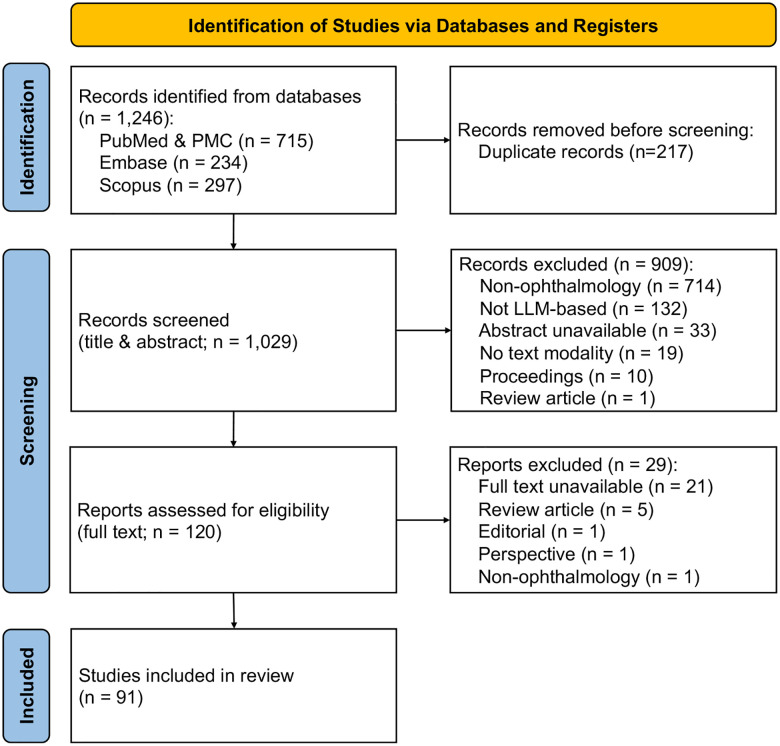
PRISMA flow diagram of study selection. The study selection process is summarized using an adapted PRISMA 2020 flow diagram^39^. A total of 1,246 records were identified across PubMed/PMC (n = 715), Embase (n = 234), and Scopus (n = 297). After removing 217 duplicates, 1,029 records underwent title and abstract screening, of which 909 were excluded. Among 120 full-text reports assessed for eligibility, 29 were excluded for reasons such as full text not available, non-ophthalmology scope, or non-research article types. Ultimately, 91 studies met the inclusion criteria and were included in the final review.

Fig. 2 presents the PRISMA flowchart outlining the study screening and selection process. Our initial literature search yielded a total of 1, 246 articles. After removing duplicate records, 1, 029 articles remained for screening. Title and abstract screening resulted in the selection of 120 articles for full-text review. During the full-text review stage, 29 articles were excluded for not meeting the inclusion criteria, leaving a final set of 91 studies included in the review.

Next, we present an integrated overview of the ophthalmic LLM literature, synthesizing key patterns across the extracted variables and providing detailed analyses and interpretations. Extended Data Table 2 presents aggregated statistics and representative top values for selected variables, serving as illustrative summaries of the full dataset. The complete, study-level dataset of extracted variables is provided in the supplementary file (please see “Data/Code Availability”).

### 3.2 Publication Landscape of Ophthalmology LLM Research

Among the 91 included studies, more than 80 different institutions served as the lead organization, indicating that ophthalmology-focused LLM research is being pursued by a diverse set of groups. Extended Data Table 2 shows Ankara Etlik City Hospital and the University of Tennessee Health Science Center led the most studies (each *n* = 5, 5.5%), followed by UC San Francisco, the University of Montreal, and the University of Toronto (each *n* = 3, 3.3%). Across countries, a total of 20 nations were represented. The United States accounted for the largest proportion (*n* = 34, 37.4%), followed by China and Turkey (each *n* = 11, 12.1%). Canada and the United Kingdom contributed seven studies, respectively (each *n* = 7, 7.7%).

Most papers were published in ophthalmology-focused or medical informatics journals. The most common venues (each *n* = 7, 7.7%) were Ophthalmology Science, Journal of Medical Internet Research, and the Cureus Journal of Medical Science, followed by the British Journal of Ophthalmology (*n* = 6, 6.6%).

Next, studies were organized by final publication date into half-year periods (H1 and H2), spanning from H1 2023 to H1 2025. The number of publications increased steadily through 2023 and 2024. Five studies were published in the first half of 2023 (H1 2023), rising to 14 in the second half (H2 2023). Growth accelerated further in 2024, with 25 studies in H1 (27.5%) and 33 in H2 (36.3%). A modest decline was observed in H1 2025 (13 studies, 14.3%), potentially reflecting the stabilization of the initial surge triggered by the release of ChatGPT and other large general-purpose LLMs, as the field transitions from early exploratory analyses to more rigorous and systematic research programs.

To characterize the nature of research contributions, we categorized studies based on whether they primarily evaluated existing LLMs or introduced ophthalmology-specific methodological adaptations or model developments (e.g., domain-specific fine-tuning or specialized model design). With respect to study aim, the overwhelming majority of publications focused on evaluating or applying existing LLMs (*n* = 82, 90.1%), whereas only nine studies (9.9%) proposed methodological contributions. Regarding input modality (i.e., whether studies used text alone or combined text with imaging data), most studies relied on text-only models (*n* = 70, 76.9%), while 21 studies (23.1%) incorporated multimodal inputs.

**Figure 3:**
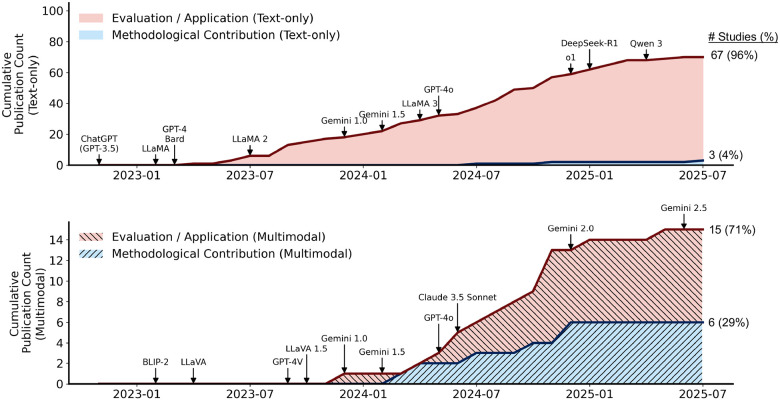
Growth of medical LLM publications over time, separated by modality and study aim. Cumulative publication counts are shown for text-only studies (top) and multimodal studies (bottom), stratified into Evaluation / Application (red) and Methodological Contribution (blue). Key model releases (e.g., ChatGPT, LLaMA series) are annotated to illustrate temporal alignment between major model introductions and subsequent increases in publication volume. The text-only literature expanded rapidly beginning in early 2023 after GPT-3.5 and GPT-4, while multimodal research accelerated later, following the release of prominent vision-language models such as LLaVA and GPT-4V. Note that publication dates reflect final journal publication; studies were included based on their availability within the predefined search window.

Fig. 3 provides a more fine-grained view, illustrating publication trends stratified by study aim and input modality. Research on text-only models expanded rapidly after the release of GPT-3.5 and GPT-4^40^, reflecting the accessibility of these models for clinical text applications. In contrast, multimodal LLM studies emerged later, aligning with the introduction of LLaVA^41^ and GPT-4V, and became more prominent in 2024 as interest in image-aware or vision-language capabilities grew within ophthalmology. Evaluation and application studies drove the initial growth, with methodological contributions emerging later. Notably, methodological contributions were scarce in text-only studies (*n* = 3, 4%) but more prevalent in multimodal research (*n* = 6, 29%).

### 3.3 Model Development and Adaptation

**Figure 4:**
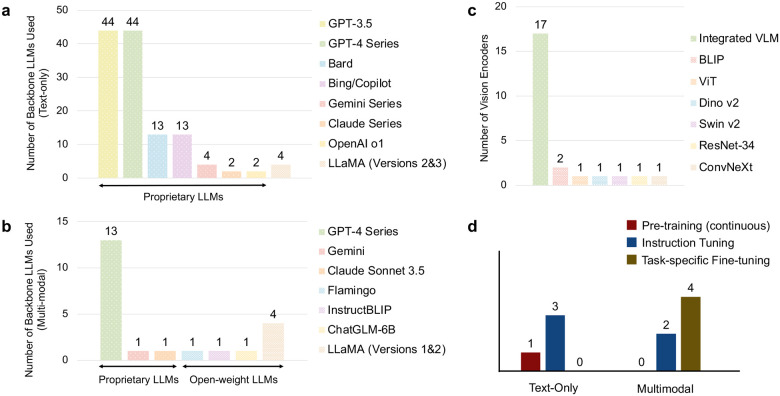
Backbone models, vision encoders, and training strategies used in LLM studies. Distributions are shown for text-only and multimodal backbone LLMs (a, b), vision encoders used in multimodal systems (c), and model training or adaptation strategies (d). Integrated VLM refers to models in which visual and language components are jointly designed and trained as a unified backbone. Numbers indicate the number of studies.

Fig. 4a–b shows the distribution of backbone LLMs used in text-only and multimodal studies, respectively. Among the 70 text-only studies, OpenAI’s GPT-3.5 and GPT-4 series were by far the most widely used models (*n* = 44, 62.9%) (Fig. 4a). Google’s Bard and Microsoft’s Bing (later rebranded as Copilot), which were released around the same period, also appeared in several studies (*n* = 13, 18.6%). These models were typically evaluated alongside ChatGPT (GPT-3.5 or GPT-4) rather than used independently, often serving as comparative systems in ophthalmology-focused tasks or case studies. More recently introduced models, such as Google’s Gemini series (*n* = 4) and OpenAI’s o1 (*n* = 2), were used less frequently. Overall, the landscape is heavily dominated by proprietary LLMs, with only a small number of studies employing open-source alternatives. Notably, just four studies used the LLaMA 2 or LLaMA 3 families.

Similarly, in the 21 multimodal studies, OpenAI models again dominated, although the pattern differed from that observed in text-only research. As shown in Fig. 4b, the GPT-4 series accounted for the largest share (*n* = 13, 61.9%), driven primarily by the use of GPT-4V for handling image inputs. Multimodal variants of other proprietary models, such as Gemini and Claude, appeared only once each. Compared with text-only studies, multimodal research showed a higher proportion of open-source models. Several studies employed open-source multimodal systems such as Flamingo^50^, InstructBLIP^51^, and ChatGLM^52^. In addition, LLaMA-based models were used in four studies, typically by combining a LLaMA backbone with a vision encoder, often following design principles similar to those used in LLaVA.

Fig. 4c shows the vision encoders used in multimodal systems. Many studies (*n* = 17, 81.0%) adopt integrated vision–language models (VLMs), in which visual and language components are jointly designed and trained as a unified backbone (e.g., GPT-4V and Gemini). Unlike language backbones, there is no single dominant vision encoder; instead, a wide variety of architectures are used across studies, including BLIP^53^, Vision Transformer (ViT)^54^, DINOv2^55^, Swin Transformer v2 (Swin v2)^56^, ResNet-34^57^, and ConvNeXt^58^.

Fig. 4d summarizes the training approaches applied to both text-only and multimodal models. For text-only studies, all three models were instruction-tuned, with one model first undergoing continuous pre-training on ophthalmic corpora before instruction tuning^42^. Multimodal models were generally trained via task-specific fine-tuning, meaning that they were trained using datasets tailored to the target downstream tasks and evaluated on corresponding test sets. One study additionally performed visual instruction tuning using ophthalmic image–text pairs^23^.

In Table 1, we summarize studies that explicitly trained or adapted models for ophthalmology specific applications, together with their corresponding methodological choices. For text-only models, most studies relied on instruction tuning, using diverse ophthalmology-related instruction–response pairs to better adapt general-purpose open-source LLMs to the domain. LLaMA-2–7B was the most commonly used backbone^42, 43^, while one study adopted ChatGLM to develop a Chinese-specialized model, leveraging its pretraining on both Chinese and English corpora^44^. For multimodal approaches, the dominant strategy was to augment text-only LLMs with visual understanding by integrating an external vision encoder, such as ViT, BLIP, and Swin v2, and fusing it with the language model. Alignment between the vision and language components was achieved through instruction tuning or task-specific training data. In contrast, one study employed an integrated vision–language model pretrained on multimodal data^49^, such as Flamingo.

### 3.4 Evaluation Task Categories

The following section describes the task areas in which the studies evaluated or developed the models. We manually normalized the initial free-text descriptions provided by annotators for the *Task Category* variable. After consolidation, the tasks were organized into three major categories (Extended Data Table 2). The three major categories were as follows: (1) Clinical workflow (*n* = 40, 44.0%), which includes applications situated within clinical practice, such as diagnostic support, treatment recommendations; (2) Patient support (*n* = 20, 22.0%), referring to patient-facing tasks such as question answering, education, and discharge-related summaries; and (3) Education and training (*n* = 31, 34.1%), involving the use of LLMs for ophthalmology board preparation or general knowledge enhancement.

These three major categories were further divided into nine fine-grained subcategories. Within the clinical workflow category, three subcategories were identified. (1) *Screening or diagnosis* (*n* = 30, 33.0%) refers to tasks in which LLMs were used to detect, classify, or reason about ocular diseases. This was the most evaluated task overall. (2) *Treatment planning and recommendation* (*n* = 10, 11.0%) includes tasks where models suggest management strategies, follow-up plans, or therapeutic options. (3) *Report generation* (*n* = 3, 3.3%) involves automated drafting or summarizing of clinical documents such as imaging reports or progress notes. This task category was evaluated in relatively few studies.

The patient support category consisted of four subcategories. (4) *Patient question answering* (*n* = 13, 14.3%) includes models responding to patient queries, often in settings resembling online Q&A platforms or patient portals. (5) *Patient education material generation* (*n* = 6, 6.6%) refers to the creation of layperson-friendly educational or discharge materials. (6) *Consultation or interview* (*n* = 1, 1.1%) involves conversational exchanges mimicking clinical interviews. (7) *Physician recommendation* (*n* = 1, 1.1%) includes tasks in which models recommend appropriate specialists or care pathways. The latter two tasks represent more specific, patient-facing applications and have been evaluated in only a small number of studies.

The education and training category included: (8) *Exam taking* (*n* = 27, 29.7%) refers to tasks in which LLMs answer ophthalmology board-style or standardized examination questions. This task was the second most frequently evaluated overall. (9) *Medical education and learning support* (*n* = 4, 4.4%) refers to tasks involving tutoring, explanation generation, or content review to support trainees or students in the medical domain. Note that these two tasks are primarily non-clinical in nature, as they do not involve real-world patient data or direct clinical decision-making.

### 3.5 Clinical Validation and Readiness Levels

To assess the rigor with which the included studies evaluated their models, we categorized each work according to its level of clinical validation and readiness across five defined stages. These stages represent a progressive trajectory from technical feasibility to real-world clinical deployment: (1) Benchmark evaluation establishes baseline algorithmic performance using standardized, retrospective datasets, typically with automated scoring. (2) Expert evaluation incorporates qualitative assessment by clinicians or domain experts, for example to examine the medical soundness, clinical relevance, or practical interpretability of model outputs. (3) Retrospective clinical validation evaluates models using historical real-world clinical data, allowing assessment under more heterogeneous and context-rich conditions. (4) Prospective pilot studies explore model performance within active or simulated clinical workflows, such as assessing real-time feasibility, safety considerations, or workflow compatibility at a limited scale. (5) Full clinical trials represent the highest level of validation, in which models are evaluated in large-scale, controlled study settings to formally assess clinical benefit, safety, or patient-level impact. This hierarchy reflects increasing clinical readiness across successive stages. Early stages (i.e., benchmark and expert evaluations), primarily address internal validity and algorithmic accuracy, whereas later stages (i.e., prospective testing and clinical trials) require robustness to the variability, uncertainty, and operational constraints of real-world healthcare settings. Progression along this continuum signifies a shift from proof-of-concept systems to clinically validated interventions. Accordingly, studies situated at the higher end of this spectrum offer the strongest evidence of clinical utility, safety, and reliability, which are essential for regulatory approval and integration into routine clinical practice.

Benchmark evaluation appeared in 48 studies (52.7%). Expert evaluation was similarly common, reported in 52 studies (57.1%), and represented the most frequently used evaluation approach. In these studies, ophthalmologists or other medical professionals qualitatively assessed model outputs, addressing aspects not captured by automated metrics and often serving as a complement to benchmark-based assessments. In contrast, more clinically grounded forms of validation were comparatively rare. Retrospective clinical validation was conducted in only 9 studies (9.9%). Even fewer studies advanced to prospective pilot testing; only two studies reached this stage. Notably, no study reached the stage of a full clinical trial, underscoring a substantial gap between technical feasibility and trial-based validation of clinical efficacy under real-world conditions

**Figure 5:**
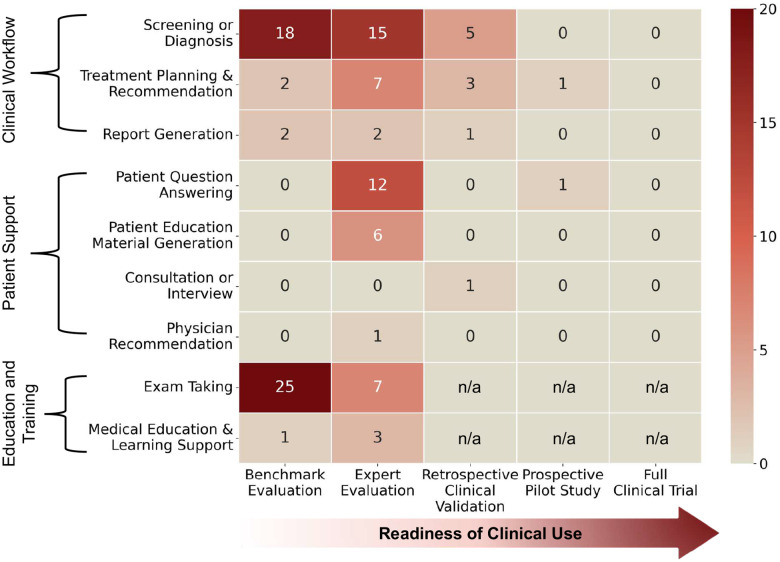
Distribution of study use cases across clinical scenarios and stages of clinical readiness. Heatmap showing the number of studies in each clinical task category (rows), mapped against increasing levels of clinical evaluation rigor (columns). A few studies advance to retrospective or prospective clinical validation, and none reach full clinical trials, highlighting the wide gap between current LLM research and real-world clinical deployment.

Fig. 5 further maps each study onto both the task categories and the corresponding evaluation depth. Note that the “exam taking” and “medical education and learning support” categories are inherently non-clinical tasks; therefore, the corresponding evaluation depth for clinical validation was marked as not applicable (n/a). Because studies often conduct more than one type of assessment, individual papers may appear in multiple validation levels. For example, a study that performed benchmark testing using standardized exam questions and additionally underwent expert review by ophthalmologists was counted in both categories.

Overall, screening and diagnosis demonstrated a wide coverage across evaluation depths, with studies spanning benchmark evaluation (*n* = 18), expert evaluation (*n* = 15), and retrospective clinical validation (*n* = 5). Treatment planning and recommendation was evaluated across the broadest range of evaluation depths, including benchmark evaluation (*n* = 2), expert evaluation (*n* = 7), retrospective clinical validation (*n* = 3), and a prospective study (*n* = 1). Similarly, report generation was evaluated primarily in benchmark (*n* = 2) and expert evaluation (*n* = 2) settings, with only a single study involving retrospective clinical validation (*n* = 1), albeit in very few studies overall.

Patient support tasks showed more varied evaluation patterns. Patient question answering was assessed primarily through expert evaluation (*n* = 12) and was the only task area that included a prospective pilot study (*n* = 1). Rather than relying on standardized benchmarks, most studies in this category used data from online patient–physician Q&A platforms, such as the American Academy of Ophthalmology (AAO) Ask an Ophthalmologist^97,98^, or manually constructed question sets^99,100^. Model outputs were subsequently evaluated by domain experts. Patient education material generation was evaluated at the benchmark (*n* = 1) and expert (*n* = 3) levels. Other patient support tasks, such as consultation or interview (*n* = 1)^101^ and physician recommendation (*n* = 1)^102^, were each represented by a single study and were evaluated through retrospective clinical validation and expert evaluation, respectively, reflecting case-based or exploratory assessments rather than standardized benchmarks.

Education- and training-oriented tasks, namely exam taking and medical education and learning support, are non-clinical and were predominantly evaluated at the benchmark level, with additional expert evaluations. Notably, exam taking had the highest number of benchmark evaluations among all task categories (*n* = 25).

### 3.6 Comparative Analysis of Model Performance

One of the key strengths of benchmark evaluation lies in its potential to allow direct comparison across models. However, in practice, results are reported across a wide range of studies, making cross-study comparisons challenging. To support a more systematic comparison of model performance, we conducted a comparative analysis of benchmark evaluation results, focusing on the two task areas most frequently assessed in the literature: exam taking and diagnosis or screening. Within these areas, we further identified the three most commonly used benchmark categories, namely (1) exam taking, (2) clinical challenges, and (3) image-based disease diagnosis, and summarize the corresponding results in the following paragraphs.

Table 2 presents results for the exam taking task. Many studies evaluated LLMs in a zero-shot setting using medical licensing and board examinations, or question banks designed for examination preparation, such as the Ophthalmic Knowledge Assessment Program (OKAP) and the Written Qualifying Examination (WQE). Some studies further included examinations used for specialty training and professional certification, including UK ophthalmology examinations administered by the Royal College of Ophthalmologists (RCOphth). Most prior evaluations in this category were limited to GPT-3.5 and GPT-4; to enable a more comprehensive and up-to-date comparison, we additionally evaluated newly introduced models, including o1^103^, GPT-5.2^104^, Gemini 3 Flash^105^, and the open-source model, gptoss-120B^106^. Across prior studies, findings were consistent in showing that GPT-4 substantially outperformed GPT-3.5, and performance was generally lower on board examination questions than on other question types, likely reflecting the greater complexity of these examinations. When considering the newly included models, we observed that advances in general-purpose model capabilities are also reflected in improved performance on ophthalmology examinations; in particular, on MedMCQA, o1, GPT-5.2, and Gemini 3 substantially outperformed both GPT-3.5 and GPT-4. Notably, Gemini 3 achieved strong performance, approaching near-perfect scores on several benchmarks, including 96% accuracy on the BCSC dataset, 97% on Multi-OphthaLingua, and 91% on MedMCQA. In addition, the open-source model gpt-oss-120B markedly outperformed the commercial GPT-4, highlighting the rapid progress of open-source LLMs.

Another line of work evaluates models using clinical challenges or quiz-style questions based on real patient queries or clinical cases (Table 3). As such, these evaluations are closer to real-world clinical scenarios and are generally more challenging than exam-style questions. The questions typically focus on diagnosis or the next step in clinical management. Similar to examination-based evaluations, prior studies in this category often relied on outdated models; thus, we included newer multimodal models in our evaluation, including GPT-4o, GPT-5.2, and Gemini 3 Flash. As shown in the table, performance improved progressively across model generations (from GPT-4V to GPT-4o and GPT-5.2), and Gemini 3 achieved the highest overall performance.

A final line of work focuses on image-based diagnostic tasks, such as binary glaucoma detection from retinal color fundus images or grading the severity of diabetic retinopathy (Table 4). Traditionally, these tasks have been addressed using supervised vision models trained on task-specific datasets. More recently, studies have explored evaluating LLMs with visual capabilities in zero- or few-shot settings, as well as lightly fine-tuning LLM-based models for these diagnostic tasks^49,76^. Among commercial models, GPT-4V has been the most commonly evaluated. A recent study has reported that Sonnet 3.5 achieves superior performance compared with GPT-4V and GPT-4o^79^. Additionally, we compared performance reported in prior LLM-based studies against the best previously reported results from non-LLM vision models. We found that non-LLM supervised models consistently achieved superior performance compared with zero- or few-shot LLM-based approaches. While fine-tuned LLM-based models achieved comparable performance on AIROGS, ODIR-2019, EyePACS, and APTOS-2019, these gains came at a substantial cost in efficiency. This raises questions about whether LLM-based approaches offer clear advantages over conventional approaches when sufficient labeled image data are available. We discuss these implications in more detail in the Discussion.

### 3.7 Resource Availability and Computational Resources

Only one third of the studies used evaluation datasets that were publicly available (*n* = 30, 33.0%). Evaluation code was available in only five studies (5.5%), a notable limitation for reproducibility given the sensitivity of LLM performance to prompting and inference parameters. Among the nine studies that conducted model training, publicly available training data were reported in four studies (*n* = 4), and training code was specified or released in four studies (*n* = 4). Compute resources were reported in the majority of studies (*n* = 8); however, training duration and total GPU-hours were less frequently reported (*n* = 4 and *n* = 2, respectively). Overall, the availability and level of detail of information on datasets and training/evaluation procedures were limited, which may in turn constrain the reproducibility of these models.

## 4 Discussion

This systematic review provides a comprehensive overview of how LLMs are currently applied, developed, and evaluated within the field of ophthalmology. By analyzing 91 studies published between January 2022 and April 2025, we systematically examined 27 variables across six analytical domains. Our findings indicate that while the field is expanding rapidly, it remains dominated by the assessment of existing general-purpose models rather than the introduction of domain-specific methodological advances. Although multimodal approaches are beginning to emerge in line with broader progress in vision–language modeling, the current landscape remains largely text-centric. Furthermore, model usage is heavily concentrated among a few widely accessible proprietary systems, such as the GPT-3.5 and GPT-4 series, with comparatively limited adoption of open-source backbones (e.g., LLaMA 2 and 3) or specialized training on ophthalmic datasets. Evaluation practices exhibit a similar imbalance; most studies rely on benchmark-style testing and expert review, whereas clinically grounded validation and prospective evaluations remain markedly uncommon. In addition, we conducted a comparative analysis of benchmark results reported in prior studies alongside evaluations of newly released models, revealing continued performance gains on multiple-choice question answering tasks, with Gemini 3 achieving particularly strong results.

### Novel insights from this review

To our knowledge, this review incorporates the most up-to-date literature and offers the broadest coverage to date among reviews of LLMs in ophthalmology (see Extended Data Table 3). Several prior reviews have surveyed the utility of LLMs and discussed their potential applications in ophthalmology^12,13,28–32^. In addition, a small number of studies have provided broader overviews of LLM evaluation across medical domains^33^. While sharing the goal of assessing the promise of LLMs and their proximity to clinical deployment, our review makes three distinct contributions.

First, we provide a methodological perspective that has been largely absent from prior reviews by explicitly distinguishing between text-only and multimodal systems. We begin by quantifying the extent to which existing studies contribute to model development and find that fewer than 10% of the included studies introduce substantive methodological innovations. This imbalance is particularly pronounced in text-only settings, where only 4% of studies propose domain-specific modeling advances (see Figure 3). Beyond documenting this gap, our review moves beyond prior work that has primarily focused on what LLMs are used for or how they are evaluated. Instead, we systematically examine how models are adapted and trained for the ophthalmology domain (see the “Model Development and Adaptation” section of the Results). Specifically, we analyze the types of models employed; the training strategies adopted—such as pre-training, instruction tuning, and task-specific fine-tuning—and, given the central role of multimodality, the vision encoders used and their integration with language models.

Second, we introduce a structured view of clinical evaluation rigor across five levels. Whereas existing reviews typically organize studies by clinical application and describe them narratively, we reframe the literature along an orthogonal axis of clinical relevance and readiness. To our knowledge, this is the first review to systematically contextualize ophthalmic LLM studies within a unified framework of clinical maturity. Applying this framework revealed a pronounced imbalance in evaluation practices across all potential LLM use cases (see “Clinical Validation and Readiness Levels” in the Results). Benchmark-based assessments (52.7%) and expert evaluations (57.1%) were common, whereas more clinically grounded forms of validation were rare, including retrospective validation using real-world data (9.9%) and prospective evaluation (2.2%). Notably, no study advanced to a full clinical trial, underscoring a substantial gap between current evaluation practices and clinically relevant validation.

Finally, in contrast to prior reviews, we explicitly examined resource availability across studies, including the public release of training and evaluation data, code, and models (see “Resource Availability and Computational Resources” in the Results). We consider this aspect critical because reproducibility and transparency are especially important in high-stakes domains such as medicine, where rigorous validation is required. Moreover, open and transparent reporting of resources is essential not only for trustworthy evaluation but also for enabling rapid and cumulative progress in the field. We found that only 33.0% of the included studies relied on publicly available evaluation datasets, and evaluation code was released in just five studies (5.5%). Among studies that involved model training, most reported basic hardware specifications, such as GPU type, but critical details required for reproducibility, including training code, training data, training duration, and model licensing, were frequently missing.

### Current state of evaluation and its maturity

Current ophthalmology LLM research remains largely anchored in an exploratory phase, particularly regarding clinical validation. When categorized by the five stages of evaluation, the vast majority of studies are confined to benchmark evaluations and expert reviews, while research dedicated to clinical validation remains critically scarce. Although benchmarking is an essential first step that provides a necessary baseline for initial testing, it cannot guarantee a model’s real-world effectiveness. This discrepancy arises primarily because benchmarks often fail to capture the dynamic variables and multifaceted decision-making processes present in actual ophthalmic practice. Furthermore, there is often a significant distributional shift between the curated, high-quality datasets used in benchmarks and the noisy data encountered in clinical environments. For example, one study included in our review evaluated ChatGPT models for glaucoma diagnosis using retrospective patient data, where model inputs consisted of structured clinical variables spanning multiple modalities and measurements, conditions that differ markedly from standardized benchmark datasets^107^. Also, real-world ophthalmic data frequently involves incomplete electronic health records, varied imaging qualities from different devices, and diverse patient demographics that are rarely fully represented in standardized testbeds. Consequently, a model that excels on a clean benchmark may experience a substantial performance degradation when faced with the unpredictability and data heterogeneity of a live clinical workflow. Moving beyond static evaluations toward rigorous clinical validation is therefore imperative to bridge the gap between technical potential and practical utility.

In addition, our review indicates that current benchmarking efforts are heavily skewed toward “diagnosis and screening” and “medical examination” tasks (Figure 5). In contrast, only two studies conducted benchmark evaluations for treatment planning and recommendations, and notably, no benchmark evaluations were identified for the patient question answering task. This disparity may stem from a lack of high-quality, task-specific benchmarks or, alternatively, the inherent difficulty of quantifying free-text generation. Many researchers opt for manual qualitative case studies over automated benchmarking due to the ambiguity of existing metrics in evaluating complex linguistic outputs. Therefore, there is an urgent need to develop and validate comprehensive benchmarks that cover the full spectrum of ophthalmic care. To address concerns such as data leakage, future benchmarking frameworks should consider a model-submission paradigm, where models are evaluated on hidden server-side datasets, to ensure the integrity and clinical validity of the results.

Furthermore, a significant portion of the studies that conducted benchmark evaluations adopted an exam-taking format to assess their models. While medical licensing examinations and multiple-choice questions have historically served as accessible testbeds for model evaluation, there is growing scholarly consensus regarding their fundamental limitations^108^. Recent research suggests that these formats may lead to an overestimation of a model’s true capabilities; even when an LLM’s underlying medical knowledge or reasoning is flawed, the model may still identify the most statistically probable answer^109^. As illustrated in Table 2, the performance of current LLMs on these standardized exams is now approaching a ceiling, suggesting that such metrics no longer provide sufficient granularity to differentiate model quality. More importantly, these exam-style assessments differ substantially from the complexity, uncertainty, and contextual reasoning required in actual ophthalmic workflows. High scores on structured examinations offer limited insight into how a model will behave in clinically realistic scenarios involving ambiguous patient presentations. Consequently, a heavy reliance on exam-based benchmarking risks overstating a model’s clinical readiness. This highlights an urgent need for evaluation frameworks that more directly reflect real-world ophthalmology practice, incorporating clinically grounded validation and workflow-relevant testing.

### Where might clinical translation be plausible?

A central practical question is which ophthalmology LLM applications appear closest to real-world clinical adoption. Importantly, we do not argue that any current system is “ready for adoption”; instead, this section highlights applications that have begun to show early empirical evidence of practical feasibility, which we consider relatively more promising for future clinical adoption.

AI-assisted treatment planning and recommendation represents one of the more promising application areas. As shown in Fig. 5, this category spans the widest range of evaluation maturity, from benchmark evaluations to prospective clinical studies, with several studies reporting alignment with expert judgments and largely appropriate responses^110,111^. However, this promise should be interpreted cautiously and in light of two key considerations. First, LLMs should be positioned as collaborative decision-support tools working alongside clinicians, rather than as standalone systems, as clinician involvement is critical for maintaining accuracy and safety. Second, models should be trained for the target task using domain-specific clinical data, rather than relying on purely zero-shot use of general-purpose LLMs. A representative example is DeepDR-LLM^45^, which was explicitly developed under these principles by learning from real-world clinical data and corresponding management recommendations. In this study, model-generated recommendations were broadly comparable to those of primary care physicians (PCPs) on real patient cases. In a prospective comparison between PCP-only workflows and workflows augmented with model recommendations, clinicians rated the LLM-supported setting favorably across multiple dimensions, including overall satisfaction, time efficiency, and perceived effectiveness. AI-supported recommendations were further associated with improved patient self-management behaviors, a 19.34% increase in ophthalmology visits within two weeks among referable diabetic retinopathy patients, and an approximately three-day reduction in time to appointment. While PCP-only guidance was often rule-based and insufficiently personalized, the PCP+AI recommendations were perceived as more personalized, higher in quality, and more empathetic. The authors suggest that these differences may have contributed to improved patient engagement and adherence.

Recent studies suggest that LLMs may serve as promising tools for patient education and Q&A. One study evaluated a GPT-4-based educational tool for patients with age-related macular degeneration (AMD) and reported high usability and user satisfaction^24^. Another study compared ophthalmology trainees and GPT-4 on patient case scenarios, finding that GPT-4 performed comparably to trainees in terms of diagnostic suggestions and triage urgency, which may support the potential role of such systems in prompting patient action and facilitating timely access to care^112^. Additional work has shown that GPT-3.5 can automatically generate patient handouts in neuro-ophthalmology at a level that specialists generally found acceptable^113^. At the same time, many studies report that chatbot-generated responses tend to have higher reading difficulty than clinician-authored or standard patient education materials. These limitations could be partially mitigated through prompt tuning, as demonstrated in prior work, by explicitly instructing the model to respond at a sixth-grade comprehension level^114^. They could be further addressed through dedicated training in future systems.

By contrast, in the areas of screening and diagnosis, substantial methodological development and more rigorous clinical validation of LLMs are still required. While some studies using public benchmarks have reported early signs of potential, evaluations conducted on real-world patient data have generally demonstrated insufficient performance for clinical deployment. For example, one study reported a diagnostic accuracy of only 31.3% for GPT-4V^115^, while another found the sensitivity of GPT-4 to be approximately 61%^107^. Such results fall short of the high reliability and safety thresholds required in clinical practice. Moreover, as summarized in Table 4, when labeled training data are available, LLM-based approaches often perform substantially worse than conventional supervised classification models. This suggests that, at present, training task-specific classifiers may be a more effective and efficient solution than applying general-purpose LLMs in zero- or few-shot settings. Although a key potential advantage of LLMs lies in their ability to integrate multimodal information and process long-context inputs, evidence from the studies analyzed in this review remains insufficient to conclude that these capabilities have translated into meaningful performance gains for screening or diagnostic tasks.

### Need for ophthalmology-specific model development

A greater emphasis on domain-specific model development is essential for the advancement of the field. Our review found that a substantial proportion of studies (over 85%) relied on proprietary models, such as GPT-3.5 and GPT-4. However, continued reliance on these off-the-shelf, closed-source systems introduces fundamental limitations for clinical translation. Because these models typically necessitate data transmission via external APIs, they often cannot be deployed on sensitive clinical data due to stringent privacy regulations and institutional security protocols^116–118^. This challenge underscores the critical importance of sustained efforts to develop on-premises models that can be hosted on secure internal servers, ensuring data sovereignty. Furthermore, general-purpose models without domain-specific training or alignment may fail to capture the intricate clinical nuances and unique distributional characteristics inherent in ophthalmic text and imaging data^119,120^. This observation aligns with prior literature suggesting that general-purpose LLMs may underperform in highly specialized fields and derive significant benefits from domain-specific adaptation^119,120^. Our analysis empirically supports this trend; as demonstrated in Table 4, models fine-tuned on ophthalmology-specific datasets substantially outperformed their unfine-tuned, general-purpose counterparts.

Furthermore, while several studies have attempted domain adaptation, their methodological frameworks exhibit notable limitations. Our analysis reveals that a significant majority of existing research relies on relatively outdated backbone architectures, such as LLaMA, LLaMA 2, or ChatGLM (see Table 1), which were widely adopted at the time many of these studies were conducted. As a result, it remains uncertain whether the proposed methods would exhibit comparable performance when integrated with more recent, state-of-the-art models that have since emerged. Beyond model selection, there are critical gaps in current training paradigms. Most studies focus primarily on standard instruction tuning to improve task adherence^42–44^, leaving advanced techniques, such as reinforcement learning for reasoningcentric model development, largely understudied in the ophthalmic context^121–123^. Future research must, therefore, move beyond simple fine-tuning and actively incorporate cutting-edge methodologies to address the complex clinical reasoning required in specialized medical domains.

### Reproducibility and transparency challenges

We identified several substantial reproducibility challenges that stem from prevailing research practices in the literature. First, many commonly used benchmark datasets are not publicly accessible and are therefore difficult to reproduce. As shown in Tables 2 and 3, examination-based evaluations and clinical challenge questions represent the most frequently studied task types. However, these datasets are often subject to licensing restrictions: while they may be used for internal LLM evaluation, they cannot be publicly shared, resulting in non-identical benchmarks across studies. For example, the Basic and Clinical Science Course (BCSC) dataset is an educational question–answer resource provided by the American Academy of Ophthalmology (AAO). Although AAO permits the use of up to 260 questions for LLM evaluation, prior studies typically do not specify which exact questions were selected. Consequently, even when multiple studies report evaluating models on “260 BCSC questions,” the underlying question sets may differ substantially, undermining comparability. Similar issues arise in studies that rely on proprietary question banks such as OphthoQuestions, StatPearls, or AMBOSS: although these sources are often cited, insufficient detail is provided regarding question selection or preprocessing, rendering independent replication infeasible.

Second, many studies fail to clearly specify the exact model checkpoints or versions used for evaluation. For instance, models are frequently reported simply as “ChatGPT-4,” despite the fact that different checkpoints or version updates may exist over time, potentially leading to performance variation. Similarly, among studies that further fine-tune open-source models, some do not report the model size (i.e., number of parameters) or clarify whether the evaluated model corresponds to a base, chat-tuned, or instruction-tuned variant, making it difficult to identify the exact model configuration used. In addition, none of these studies release their training code or clearly specify the training framework or implementation employed. The lack of precise model versioning and training transparency further complicates cross-study comparison and undermines reproducibility.

Third, transparency regarding evaluation protocols remains limited (see “Resource Availability and Computational Resources” in the Results). Exact prompts, inference settings, and evaluation code are often omitted, and only a small fraction of studies release their evaluation scripts. Without access to these components, reproducing reported results or conducting fair comparisons becomes challenging, even when similar datasets and models are used.

### Limitations

Our study has several limitations. First, our literature search was conducted using PubMed, Scopus, and Embase. While these databases are widely used and well suited for biomedical research, they may not comprehensively capture relevant work from the AI or computer science literature, including preprints hosted on platforms such as arXiv. To address this, and as noted in the Introduction, we will maintain a dedicated website to complement this review. This platform will facilitate ongoing supplementary searches to identify missed studies and allow for continuous updates in collaboration with the research community.

Second, the inherent time-lag in systematic reviews poses a significant challenge in rapidly evolving fields. By their nature, systematic reviews capture a specific window of time, and additional time inevitably elapses while the collected data is being rigorously screened, analyzed, and synthesized. This process creates an unavoidable gap between the final search date and the point of publication. Although our search timeline from January 2022 to April 2025 provides the most current and comprehensive synthesis available at this time as shown in Extended Data Table 3, the fast-paced nature of AI research means that significant developments may emerge during these intervening months. To mitigate this, the dedicated website mentioned above will serve as a platform for a “living” version of this review. We are committed to providing continuous updates, ensuring that this resource remains relevant. We believe this ongoing effort is crucial to maintaining the review as a functional tool that keeps pace with the field as it evolves.

Third, although our framework offers a structured view of clinical maturity, it does not directly address model safety characteristics. Key considerations for real-world use, including hallucinations, algorithmic bias, and explainability, were therefore outside the scope of this review. This reflects a deliberate focus on translational progress: instead of emphasizing individual performance scores, we examined how existing studies assess and validate models along the pathway from benchmarks to clinical testing. While quantitative measures remain relevant, we prioritized the level of evaluation as a more meaningful signal of practical readiness. Future work could extend this perspective by integrating evaluation rigor with standardized safety assessments, yielding a more complete picture of LLM preparedness for clinical use.

## Extended Data

**Extended Data Table 1:** Data extraction and annotation guidelines for included studies.

Feature	Annotation Guideline
**Publication Metadata**	
*Lead Institution*	List the institution of either the first author or the corresponding author (you may include multiple if applicable).
*Country of Lead Institution*	Identify the country of the institution listed under “Lead Institution.” Use the country where the institution is physically located. If multiple lead institutions are provided, list all corresponding countries.
*Publication Venue*	Specify the publication venue, such as the journal name, conference name, or preprint server. Use the official full name of the venue when available (e.g., Nature Medicine, JAMA Ophthalmology, arXiv).
*Date of Publication*	Record the publication date of the study, using year and month only (YYYY-MM). For journal articles, follow the official publication month provided by the venue (either online publication month or issue month). For preprints, use the posted month.
**Scope & Purpose**	
*Study Aim*	(1) Application: Evaluating existing models on ophthalmology tasks or applying a general LLM to ophthalmology without proposing a new model (2) Methodological contribution: Introducing a new ophthalmology-specific model, architecture, a new training strategy, alignment method, or adaptation technique, etc.
*Subspecialty*	Specify the ophthalmic subspecialty addressed by the study (e.g., retina, glaucoma, cornea, oculoplastics, neuro-ophthalmology).
*Input Modality*	Indicate the type(s) of input data used by the model. Options: (1) Text-only (2) Multimodal
**Model Architecture & Training**	
*Model Adaptation*	Indicate the model adaptation applied in the study: (1) Pre-training (from scratch), (2) Pre-training (continuous), (3) Instruction tuning, (4) Task-specific fine-tuning, (5) Preference alignment, and (6) Training-free.
*Base LLM*	Underlying language model used (e.g., Llama 3, GPT-4)
*Number of Parameters*	Total model size, typically reported in billions (e.g., 7B, 34B, 70B).
*Vision Encoder*	Image encoder used (e.g., BLIP, ViT-L/14, SigLIP).
**Evaluation & Validation**	
*Task Category*	Indicate the evaluation tasks addressed in the study (e.g., Diagnosis, Screening, Prognosis or risk prediction, Treatment planning or recommendation, Triage, Clinical documentation, Counseling or patient communication, Patient education, Administrative support).
*Evaluation Depth*	Indicate the highest level of evaluation rigor employed, such as benchmark evaluation, expert review, retrospective clinical validation, prospective pilot testing, or clinical trial.
*Dataset Names*	List the names of datasets used for evaluation. Use official dataset names when available.
*Sample Size*	Report the number of samples used for evaluation (e.g., number of questions, images, cases, or patients). If multiple datasets are used, list sample sizes separately.
*Metrics*	List all evaluation metrics reported in the study (e.g., accuracy, AUROC, F1 score, ROUGE, expert rating scales).
*Comparators*	Specify the baseline or comparator models used for performance comparison (e.g., GPT-3.5, human experts, prior state-of-the-art models).
*Statistical Validation*	Indicate whether statistical analyses were conducted to support comparisons. Yes or No.
**Resource Availability**	
*Model Status*	Classify the model availability into one of the following categories: (1) API-only (model accessible only through a hosted API, with no released weights), (2) Open-weight (model weights are publicly released for download and local use), or (3) Not available (model is neither accessible via API nor released).
*Model License*	Specify the license under which the model is released (e.g., Apache 2.0, MIT).
*Evaluation Data Availability*	Specify whether the evaluation data is publicly available. Yes or No.
*Evaluation Code Availability*	Specify whether the evaluation code is publicly available. Yes or No.
*Training Data Availability*	Specify whether the training data is publicly available. Yes or No.
*Training Code Availability*	Specify whether the training code is publicly available. Yes or No.
**Compute Resources**	
*Compute Environment*	Describe the reported compute environment, including hardware type if available (e.g., GPU model, number of GPUs, cloud or institutional cluster).
*Training Duration*	Report the total training or fine-tuning time (e.g., hours or days).
*Total GPU-hours*	Record the total GPU-hours consumed if explicitly stated or directly inferable from the reported information.

**Extended Data Table 2:** Summary of key study characteristics.

Variable	Top Values	Number of Studies (%)
**Publication Metadata**		
*Lead Institution*	Ankara Etlik City Hospital, University of Tennessee Health Science Center	Each *n* = 5 (5.5%)
	UC San Francisco, University of Montreal, University of Toronto	Each *n* = 3 (3.3%)
*Country of Lead Institution*	USA	*n* = 34 (37.4%)
	China, Turkey	Each *n* = 11 (12.1%)
	Canada, UK	Each *n* = 7 (7.7%)
*Publication Venue*	Ophthalmology Science, Journal of Medical Internet Research, Cureus Journal of Medical Science	Each *n* = 7 (7.7%)
	British Journal of Ophthalmology	*n* = 6 (6.6%)
*Date of Publication*	H2 2024	*n* = 33 (36.3%)
	H1 2024	*n* = 25 (27.5%)
	H2 2023	*n* = 14 (15.4%)
	H1 2025	*n* = 13 (14.3%)
	H1 2023	*n* = 5 (5.5%)
**Scope & Purpose**		
*Study Aim*	Evaluation/Application	*n* = 82 (90.1%)
	Methodological Contribution	*n* = 9 (9.9%)
*Subspecialty*	General Ophthalmology or Not specified	*n* = 44 (48.4%)
	Glaucoma, Retina & Vitreous	Each *n* = 8 (8.8%)
	Cornea & External Disease	*n* = 5 (5.5%)
*Input Modality*	Text-only	*n* = 70 (76.9%)
	Multimodal	*n* = 21 (23.1%)
**Model Architecture & Training**		
*Model Adaptation*	Pre-training (from scratch)	*n* = 0
	Pre-training (continuous)	*n* = 1
	Instruction tuning	*n* = 4
	Task-specific fine-tuning	*n* = 4
	Preference alignment	*n* = 0
	Training-free	*n* = 0
*Base LLM*	GPT-4 series	*n* = 57 (62.6%)
	GPT-3.5	*n* = 44 (48.4%)
	Gemini series	*n* = 14 (15.4%)
	Bard, Bing/Copilot	Each *n* = 13 (14.3%)
*Number of Parameters*	Not reported	*n* = 83 (91.2%)
	7B	*n* = 5 (5.5%)
	6B	*n* = 2 (2.2%)
	9B, 80B	Each *n* = 1 (1.1%)
*Vision Encoder*	GPT-4v/−4o	n=13
	BLIP	n=2
	Gemini, Claude Sonnet 3.5, Flamingo, InstructBLIP ViT, Dino v2, Swin v2, ResNet-34, ConvNeXt	Each *n* = 1
**Evaluation & Validation**		
*Task Category*	*Clinical workflow* (*n* = 40,44.0%)	
	Screening or diagnosis	*n* = 30 (33.0%)
	Treatment planning and recommendation	*n* = 10 (11.0%)
	Report generation	*n* = 3 (3.3%)
	*Patient support* (*n* = 20,22.0%)	
	Patient question answering	*n* = 13 (14.3%)
	Patient education material generation	*n* = 6 (6.6%)
	Consultation or interview	*n* = 1 (1.1%)
	Physician recommendation	*n* = 1 (1.1%)
	*Education and training* (*n* = 31,34.1%)	
	Exam taking	*n* = 27 (29.7%)
	Medical education and learning support	*n* = 4 (4.4%)
*Evaluation Depth*	Benchmark Evaluation	*n* = 48 (52.7%)
	Expert Evaluation	*n* = 52 (57.1%)
	Retrospective Clinical Validation	*n* = 9 (9.9%)
	Prospective Pilot Study	*n* = 2 (2.2%)
	Full Clinical Trial	*n* = 0
*Dataset Names*	See the supplementary file for the full data.	
*Sample Size*	See the supplementary file for the full data.	
*Metrics*	See the supplementary file for the full data.	
*Comparators*	See the supplementary file for the full data.	
*Statistical Validation*	Yes	*n* = 69 (75.8%)
	No	*n* = 22 (24.2%)
**Resource Availability**		
*Model Status*	API-only	*n* = 79 (86.8%)
	Open-weight	*n* = 4 (4.4%)
	Not available	*n* = 8 (8.8%)
*Model License*	Apache 2.0, MIT License	Each *n* = 1
*Evaluation Data Availability*	Yes	*n* = 30 (33.0%)
	No	*n* = 61 (67.0%)
*Evaluation Code Availability*	Yes	*n* = 5 (5.5%)
	No	*n* = 86 (94.5%)
*Training Data Availability*	Yes	*n* = 4
	No	*n* = 5
*Training Code Availability*	Yes	*n* = 4
	No	*n* = 5
**Compute Resources**		
*Compute Environment*	See the supplementary file for the full data.	
*Training Duration*	See the supplementary file for the full data.	
*Total GPU-hours*	See the supplementary file for the full data.	

The table reports the most frequently observed values for each variable, along with the number and proportion of studies.

**Extended Data Table 3:** Comparison of ophthalmology LLM review articles and our proposed study across key reporting dimensions.

			Scope					
Review	Search Period	# Studies	Utility or Use Cases	Study Trends	Model Adaption	Comparitive / Meta Analysis	5-level of Evaluation Rigors	Resources & Availability
Tan et al.^12^	Up to April 2023	-	✓	✗	✗	✗	✗	✗
Jin et al.^13^	Jan 2016–Jun 2023	108	✓	✗	✗	✗	✗	✗
Wong et al.^14^	Jan 2022–Jul 2023	32	✓	✓	✗	✗	✗	✗
Chotcomwongse et al.^28^	Up to Feb 2024	-	✓	✗	✗	✗	✗	✗
See et al.^29^	Jan 2019–Mar 2024	49	✓	✗	✗	✓	✗	✗
Artiaga et al.^30^	Up to Jun 2024	46	✓	✗	✗	✗	✗	✗
Agnihotri et al.^31^	Up to Jun 2024	101	✓	✗	✗	✗	✗	✗
Bellanda et al.^32^	Jan 2022–Jul 2024	68	✓	✗	✗	✗	✗	✗
Zhang et al.^33^	Up to Nov 2024	187	✓	✗	✗	✓	✗	✗
Wei et.al.^34^	Up to Mar 2025	21	✗	✗	✗	✓	✗	✗
**Ours**	Jan 2022–Apr 2025	91	✓	✓	✓	✓	✓	✓

Studies are ordered by the end date of the time period.

## Figures and Tables

**Table 1:** Summary of ophthalmology-specific large language models and vision–language models.

Model Name	Base LLM	# Params.	Vision Encoder	Model Adaptation	Key Use Cases
**Input Modality: Text-only**					
Eye-LLaMA^42^	LLaMA 2	7B	-	PT (continuous), IT	QA, Chatbot
EyeGPT^43^	LLaMA 2	7B	-	IT	QA, Chatbot
MOPH^44^	ChatGLM2	6B	-	IT	QA (Chinese)
**Input Modality: Multimodal**					
DeepDR-LLM^45^	LLaMA	7B	Vision Transformer	Task-specific	Primary diabetes care
slit lamp-GPT^46^	LLaMA 2	Unspecified	BLIP	Task-specific	Report generation, QA (Slit lamp)
FFA-GPT^47^	LLaMA 2	Unspecified	BLIP	Task-specific	Report generation, QA (FFA)
EyeGraphGPT^23^	LLaMA 2	7B	Swin Transformer v2	IT (visual alignment)	Report generation
OphGLM^48^	ChatGLM	6B	ConvNeXt	IT	QA, Chatbot (Chinese)
Han et al.^49^	Flamingo	80B, 9B	Flamingo (Vision)	Task-specific	DR detection/grading

PT: Pre-training. IT: Instruction tuning QA: Question answering. FFA: Fundus fluorescein angiography. DR: Diabetic retinopathy.

**Table 2:** Model performance on ophthalmology exam-style questions.

	Evaluated Models					
Dataset Name & Source	GPT-3.5	GPT-4	o1	GPT-5.2	Gemini 3	gpt-oss-120b
**Board Exams or Question Banks**						
BCSC Complete Set	-	-	0.93^†^	0.92^†^	0.96^†^	0.86^†^
BCSC Self-Assessment Program	0.56^59^, 0.57^60^, 0.59^61^	0.72^61^, 0.81^60^	-	-	-	-
OphthoQuestions	0.43^59^, 0.46^62^, 0.64^63^	0.78^63^	-	-	-	-
StatPearls	0.56^13^	0.73^13^	-	-	-	-
Board Exam (Brazilian)	0.47^64^	0.63^64^	-	-	-	-
Board Exam (Turkish)	-	0.54^65^	-	-	-	-
**Medical Licensing Exams or Question Banks**						
Multi-OphthaLingua^66^ (Brazilian)	-	-	0.91^†^	0.89^†^	0.97^†^	0.81^†^
MedMCQA^67^ (Indian)	0.55^42^, 0.58^68^	0.75^42^, 0.78^42^	0.88^42^	0.86^†^	0.91^†^	0.81^†^
AMBOSS - USMLE Step 1	0.75^69^	0.70^69^	-	-	-	-
AMBOSS - USMLE Step 2	0.73^69^	0.90^69^	-	-	-	-
AMBOSS - USMLE Step 3	0.61^69^	0.96^69^	-	-	-	-
**RCOphth Exams**						
FRCOphth Part 1	-	0.86^70^	-	-	-	-
FRCOphth Part 2	0.48^71^	0.69^71^	-	-	-	-

All datasets consist of multiple-choice questions, and accuracy is used as the evaluation metric.

† indicates models that are newly evaluated in this study to provide a more comprehensive comparison.

**Table 3:** Model performance on clinical challenges.

	Evaluated Models					
Dataset Name	GPT-3.5	GPT-4	GPT-4V	GPT-4o	GPT-5.2	Gemini 3
**JAMA Clinical Challenge**						
Text-only						
Diagnosis	-	0.48^72^	-	-	-	-
Next plan	-	0.63^72^	-	-	-	-
All	-	-	-	0.69^†^	0.76^†^	0.82^†^
Multimodal						
All	n/a	n/a	-	0.66^†^	0.75^†^	0.83^†^
**AAO Diagnose This**						
Text-only	0.46^73^	0.75^73^	-	-	-	-
Multimodal	n/a	n/a	0.71^74^	0.77^74^	-	-
**OCTCases**						
Multimodal	n/a	n/a	0.51^75^	-	-	-

All datasets consist of multiple-choice questions, and accuracy is used as the evaluation metric. For text-only evaluations, images were either removed from the original questions or only questions without images were used, whereas multimodal evaluations used the original multimedia questions.

† indicates models that are newly evaluated in this study to provide a more comprehensive comparison.

**Table 4:** Model performance for image-based diagnostic tasks.

		Zero- or Few-shot			Supervised		
Dataset Name (Subspecialty)	Metrics	GPT-4V	GPT-4o	Sonnet 3.5	Flamingo (80B)^49^	DINOv2 + LLaMA 2 (7B)^76^	Non-LLM SOTA
ACRIMA^77^ (Glaucoma)	Acc.	0.68^78^	0.6785^79^	0.8549^79^	-	-	0.9664^80^
	Sensitivity	0.71^78^	0.8147^79^	0.9492^79^	-	-	0.9607^80^
	Specificity	0.66^78^	0.5049^79^	0.7346^79^	-	-	0.9739^80^
ORIGA^81^ (Glaucoma)	Acc.	0.70^78^	-	-	-	-	0.9575^82^
	Sensitivity	0.76^78^	-	-	-	-	0.9490^82^
	Specificity	0.65^78^	-	-	-	-	0.9475^82^
RIM-ONE^83^ (Glaucoma)	Acc.	0.81^78^	-	-	-	-	0.9615^84^
	Sensitivity	0.92^78^	-	-	-	-	0.9785^84^
	Specificity	0.74^78^	-	-	-	-	0.9238^84^
REFUGE^85^ (Glaucoma)	Acc.	0.56^86^	-	-	-	-	0.9424^87^
	Sensitivity	0.625^86^	-	-	-	-	0.9881^87^
	Specificity	0.5543^86^	-	-	-	-	-
**AIROGS**^88^ & **ODIR-2019**^89^ (Glaucoma)							
Merged evaluation	AUC	-	-	-	0.868^49^	-	-
Separate (AIROGS)	AUC	-	-	-	-	-	0.9687^90^
Separate (ODIR-2019)	AUC	-	-	-	-	-	0.7260^91^
**EyePACS**^92^ & **APTOS-2019**^93^ (Diabetic Retinopathy)							
Merged evaluation	AUC	-	-	-	0.949^49^	-	-
Separate (EyePACS)	AUC	-	-	-	-	-	0.9270^94^
Separate (APTOS-2019)	AUC	-	-	-	-	-	0.9850^95^
BRSET^96^	Acc.	-	-	-	-	0.987^76^	-
(Multiple Retinal Diseases)	F1	-	-	-	-	0.944^76^	-

All datasets consist of retinal fundus images. The non-LLM state-of-the-art results correspond to the best-performing supervised vision models reported prior to the adoption of LLM-based approaches.

## Data Availability

The full list of included studies, along with all extracted data, is provided in https://drive.google.com/drive/folders/1lbAnbftwtngQY-eGIt1H2xJAA0LyHg5Q?usp=sharing. In addition, we maintain a publicly accessible website that hosts visual summaries, detailed search methodology, and the code used for LLM evaluation, available at https://github.com/yale-BIDS-Chen-Lab/ophtho-llm-review.
